# Nanoparticle-based drug delivery systems for the treatment of cardiovascular diseases

**DOI:** 10.3389/fphar.2022.999404

**Published:** 2022-09-12

**Authors:** Fangyu Yang, Jianjiang Xue, Guixue Wang, Qizhi Diao

**Affiliations:** ^1^ Department of Clinical Laboratory Medicine, University-Town Hospital of Chongqing Medical University, Chongqing, China; ^2^ Key Laboratory for Bio-Rheological Science and Technology of Ministry of Education, State and Local Joint Engineering Laboratory for Vascular Implants, Bioengineering College of Chongqing University, Chongqing, China; ^3^ Department of Clinical Laboratory Medicine, Sanya Women and Children’s Hospital Managed by Shanghai Children’s Medical Center, Hainan, China

**Keywords:** cardiovascular diseases, nanoparticles, drug delivery, nanomedicine, nanotoxicity

## Abstract

Cardiovascular disease is the most common health problem worldwide and remains the leading cause of morbidity and mortality. Despite recent advances in the management of cardiovascular diseases, pharmaceutical treatment remains suboptimal because of poor pharmacokinetics and high toxicity. However, since being harnessed in the cancer field for the delivery of safer and more effective chemotherapeutics, nanoparticle-based drug delivery systems have offered multiple significant therapeutic effects in treating cardiovascular diseases. Nanoparticle-based drug delivery systems alter the biodistribution of therapeutic agents through site-specific, target-oriented delivery and controlled drug release of precise medicines. Metal-, lipid-, and polymer-based nanoparticles represent ideal materials for use in cardiovascular therapeutics. New developments in the therapeutic potential of drug delivery using nanoparticles and the application of nanomedicine to cardiovascular diseases are described in this review. Furthermore, this review discusses our current understanding of the potential role of nanoparticles in metabolism and toxicity after therapeutic action, with a view to providing a safer and more effective strategy for the treatment of cardiovascular disease.

## Introduction

Heart failure, arrhythmia, atherosclerosis, coronary heart disease, myocardial infarction (MI), peripheral arterial disease, deep vein thrombosis, and inflammatory heart disease, along with other cardiovascular diseases (CVDs) are a primary cause of death worldwide. The World Heart Federation has reported that CVDs account for 17.3 million deaths per year (Smith et al., 2012). The number of deaths is expected to increase over the next 10 years because of an increased prevalence in risk factors for CVDs, such as obesity, high non-high-density lipoprotein (HDL) cholesterol levels, diabetes, hypertension, tobacco use, lack of physical activity, unhealthy diet, and expansion of the geriatric population (Iafisco et al., 2019). Deaths from CVDs are expected to reach 23.6 million each year by 2030 (Yusuf et al., 2020). Different treatments of CVDs are selected based on patient risks stratification and severity. The main purpose of all treatment protocols for CVDs is to better promote blood supply and diminish tissue damage to minimize cardiomyocyte loss and increase contractile area. Severe CVD cases are usually treated with surgery to remove blood clots, place artificial cardiac pacemakers in cases of arrhythmia, and repair the pathological organic cardiac changes. Not surprisingly, maintaining regular drug therapy is relatively difficult; in most circumstances, the treatment will have to be taken for life.

Statin therapy is recommended as first-line therapy for most patients with hypercholesterolemia. In addition to the lipid-regulating effects, statin therapy is efficacious in dissolving blood clots, fighting inflammation, and improving endothelial function (Fisher and Moonis, 2012). Aspirin is the most used drug for the secondary prevention of CVDs (Baigent et al., 2009). β-blockers are adrenergic receptor antagonists that can effectively antagonize sympathetic excitability and cardiotoxicity. Therefore, β-blockers are also prescribed as the first-line treatment of atrial fibrillation and CVDs; however, they are not suitable for patients with hypertension (Martinez-Milla et al., 2019). Angiotensin converting enzyme inhibitors and angiotensin Ⅱ receptor blockers have become the drugs of choice for the treatment of heart failure, coronary artery disease, MI, and hypertension (Regoli et al., 2012). Angiotensin II receptor blocker-neprilysin inhibitor drugs both block the activation of the renin-angiotensin-aldosterone system in patients with heart failure and inhibit the activity of enkephalinase to increase the levels of various endogenous vasoactive peptides (Owens et al., 2017). The PARADIGM-HF and PARAGON-HF trials have confirmed that sacubitril-valsartan has an established role in the treatment of patients with heart failure with reduced or preserved ejection fraction (McMurray et al., 2014; Solomon et al., 2019). Although significant progress in existing treatments has been made in the past decade, the therapeutic effects of pharmacotherapy are suboptimal because of the non-specific cytotoxicity, poor solubility and absorption, first pass metabolism, poor biocompatibility, and low bioavailability of existing cardiovascular drugs (Karunakar et al., 2016).

**FIGURE 1:**
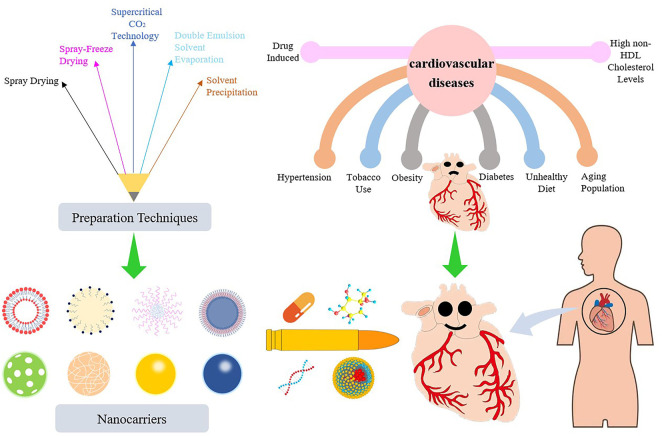
Nanoparticle-based drug delivery systems for the treatment of cardiovascular diseases.

Nanotechnology is a multidisciplinary research field involving electronics, biology, and medicine. At the end of the 19th century, the famous German bacteriologist Paul Ehrlich put forward the concept of the “magic bullet” (Valent et al., 2016). Nanomedicine or nano-biotechnology was considered one of the most active and rapid research areas of nanotechnology and had drawn worldwide attention in previous decades. Nanoparticle-based drug delivery systems (nano-DDSs) with various shapes, sizes, structures, and transport functions depend on the properties and synthesis methods of different nanomaterials, whereby ideal nanocarriers are constructed and designed (Lee et al., 2017). Nanoparticles (NPs) utilize the enhanced permeability and retention effect to precisely deliver drugs to atherosclerotic plaques, resulting in superior therapeutic effects and decreased tissue damage (Nakamura et al., 2016). In addition, nano-DDSs show considerable potential in improving drug efficacy, prolonging drug action, improving bioavailability, targeting passively or actively, reducing drug resistance, and reducing adverse drug reactions (Patra et al., 2018). In this paper, we summarize and discuss the progress of NPs as drug carriers in the treatment of CVDs and the deficiencies of nano-DDSs in clinical application. Greater emphasis is placed on NP-directed therapy for atherosclerosis and its associated complications, including arrhythmia, ventricular remodeling, MI, and myocardial ischemia-reperfusion injury (IRI), given their crucial status as CVDs (Figure 1).

### Nanoparticles

**FIGURE 2:**
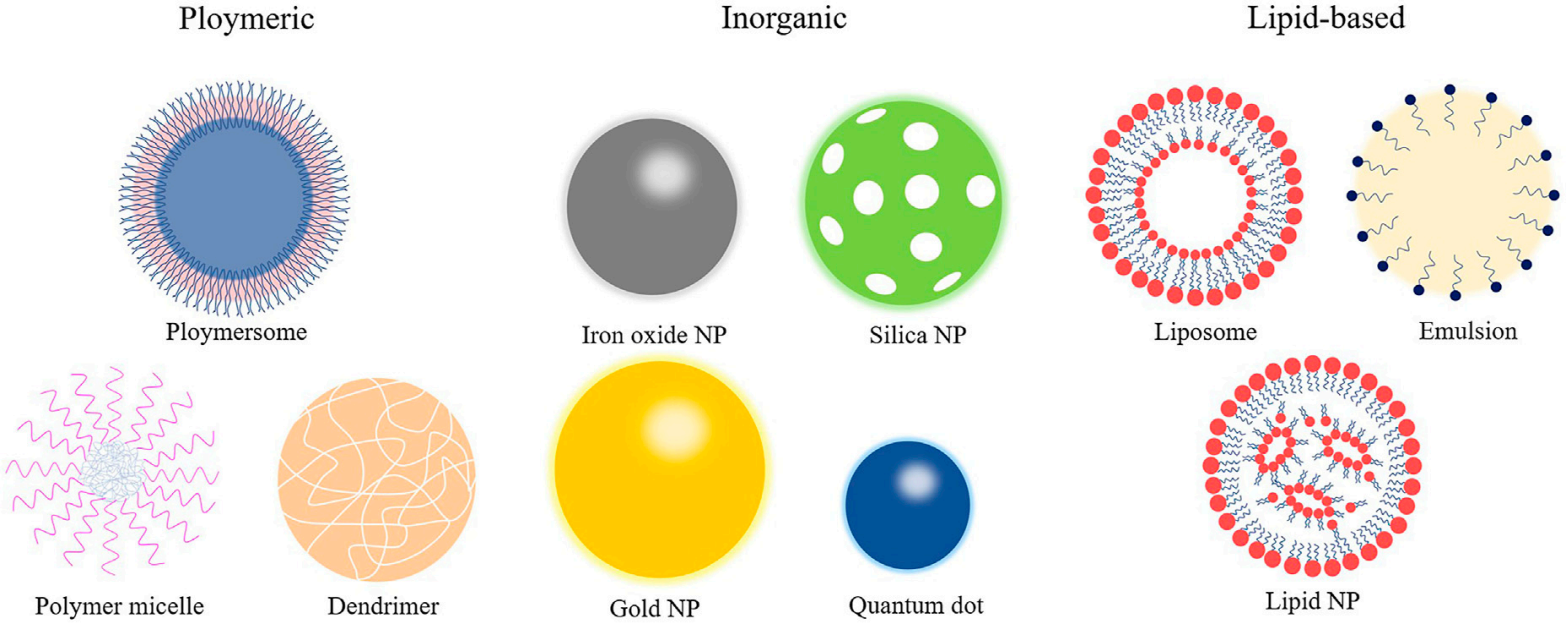
Types of nanoparticles.

NPs are organic or inorganic structures that are generally <100 nm in at least one dimension (Wang et al., 2008). Organic NPs consist of different biodegradable materials, such as lipids, liposomes or micelles, proteins, dendrimers, polymeric vesicles, or hyaluronic acid, and inorganic NPs are compounded from a variety of minute-sized structures including quantum dots, mesoporous silicon, graphene, carbon nanotubes, metals, or metal oxides (Khafaji et al., 2019). Metal-organic frameworks (also known as porous coordination polymers) comprising organic ligands and metal ions/metal clusters *via* coordinate bonds are highly porous and crystalline polymers (He et al., 2021). Metal-organic framework nanomaterial surfaces are enveloped with polyethylene glycol entirely, which reduces the clearance by the immune system (Cutrone et al., 2019). The characteristics of NPs in size and shape, interconnected macropores, tunable porosity, chemical composition, and easy surface functionalization have drawn global attention for the past few years in drug delivery research. The combination of cell carrier and nano-drug delivery technology is also becoming a hotspot because it uses the natural character of circulating cells to overcome the immunogenicity of nanomaterials (Kroll et al., 2017). For instance, NPs clothing themselves in a skin of platelet or red blood cell membranes were used to load rapamycin. It is well established that biomimetic NPs can avoid macrophage phagocytosis *in vitro*, and the therapeutic effect is superior to the traditional nano-drug delivery method *in vivo* (Wang et al., 2019; Han et al., 2022). At present, the most applicable and comprehensive nanomaterials in the diagnosis and treatment of CVDs mainly include liposomes, micelles, dendrimers, polymer NPs, and metal NPs (Figure 2). As shown in Table 1, different types of some typical nanocarriers applications for the targeted delivery of cardioprotective agents have described.

**TABLE 1:** Nanoparticles studied for the efficient treatment of cardiovascular diseases.

Types of nanoparticles	Disease targeted	Drugs used in the treatment of CVDs	Model organisms used	Biological functions	References
Lipid-based NPs					
liposomes	Atherosclerosis	Glucocorticoids	Rabbit model	Diminished the number of macrophages in the plaque and anti-angiogenic effects	Lobatto et al. (2010)
liposomes	Arrhythmia	Amiodarone	Rat model	Reduced the mortality due to lethal arrhythmia and the negative hemodynamic changes caused by amiodarone	Takahama et al. (2013)
PEGylated liposome	Myocardial infarction	Growth factors and cytokines	Cardiac cell of rats and mouse model	Delivered therapeutic agents specifically to the infarcted heart	Dvir et al. (2011)
Recombinant HDL	Atherosclerosis	Pitavastatin	Mouse model and *in vitro*	Promoted the rapid regression of plaques	Jiang et al. (2019)
Micelle-based NPs					
Modular multifunctional micelles	Atherosclerosis	Antithrombin	Mouse model	Targeted atherosclerotic plaques initially and bound to clotted plasma proteins	Peters et al. (2009)
peptide amphiphilic micelles	Atherosclerosis	microRNA-145	Mouse model	Modulated the phenotype of VSMCs to slow plaque progression	Chin et al. (2021)
Polymeric-based NPs					
PLGA	Atherosclerosis	Pitavastatin	Mouse model	Inhibited plaque destabilization and rupture by regulating MCP-1/CCR2–dependent monocyte recruitment	Katsuki et al. (2014)
Methyl-β-cyclodextrin	Atherosclerosis	Simvastatin	Mouse model	Targeted atherosclerotic plaques and reduced plaque content of cholesterol and macrophages	Kim et al. (2020)
PLGA	Pulmonary arterial hypertension	Beraprost	Rat model	Decreased pulmonary vascular resistance and inhibited pulmonary vascular remodeling	Akagi et al. (2016)
PLGA	Ischemia-reperfusion injury	Irbesartan	Mouse model	Inhibited the recruitment of inflammatory monocytes, reduced the infarct size and ameliorated left ventricular remodeling	Nakano et al. (2016)
Chitosan-alginate NPs	Myocardial infarction	Placental growth factor	Rat model	Provide sustained slow-release placental growth factor therapy	Binsalamah et al. (2011)
Dendrimer-based NPs					
Poly (amidoamine)-histidine nanocarriers	Myocardial infarction	miRNAs	H9c2	Prevented the hypoxia/reperfusion-induced apoptosis critical in myocardial infarctions	Sayed et al. (2020)
Metal-based NPs					
CuS NPs	Atherosclerosis	Antibodies	Mouse model	Reduced lipid accumulation	Gao et al. (2018)
AuNPs	Hypertension	Antibodies	*In vitro*	The detection of cortisol ranged from 0.1 to 1000 ng/ml with a detection limit of 0.05 ng/ml at 3σ	Sun et al. (2017)
Fe_3_O_4_ NPs	Thrombosis	t-PA	Swine model	Delivered t-PA to the thrombosis area and the drug accumulation at the lesion site was significantly increased	Cicha. (2015)

### Lipid-based nanoparticles

Liposomes are among the most typical subsets of lipid-based NPs, which are the monolayer and multilayer vesicles synthesized from phospholipids through complex processes (Chiani et al., 2017). This special amphiphilic structure enables liposomes to easily carry and deliver drugs with different properties, such as hydrophilicity, hydrophobicity, and lipophilicity, and even adsorb hydrophilic and lipophilic compounds at the same time (Teymouri et al., 2019). Currently, numerous liposome formulations have been developed for the prevention and treatment of atherosclerosis and its complications. Lobatto et al. (2010) devised a nano-medicinal liposomal formulation of glucocorticoids. This liposome formulation utilized the high permeability of blood vessels to transport glucocorticoids to the vulnerable plaque, which significantly diminished the number of macrophages in the plaque. There was no obvious toxicity observed, indicating that this liposome formulation could be used as a high-quality anti-atherosclerosis drug formulation.

### Micelle-based nanoparticles

Micelle-based NPs can be fashioned into various structure subpopulations; the spherical structure is most typical among them. Micelles are self-assembled from lipids or amphiphilic molecules dissolved in water. They generally consist of a hydrophilic inner core and a hydrophobic shell. The inner core is the loading space for insoluble drugs, and the shell is the protective interface between the core and the external environment (Gothwal et al., 2016). Compared with liposomes, micelles are smaller in size, more compact in spatial structure, and have a relatively lower loading capacity. Hence, the ischemic myocardium is more permeable to micelles, which are more targeted to the lesions. In addition to providing conventional drug delivery, micelles can also target specific components of plaques. Peters et al. (2009) developed a modular multifunctional micelle loaded with antithrombin that targets atherosclerotic plaques initially and binds to clotted plasma proteins. The targeted micelles delivery system observably increased the antithrombin activity in diseased vessels and reduced the risk of plaque rupture.

### Polymeric-based nanoparticles

Polymer NPs can be synthesized using a variety of natural or synthetic macromolecular materials with different structures, and the particle size and surface charge of NPs change along with the polymer type. Drugs are usually integrated into polymer NPs with various strategies, such as dissolution, encapsulation, embedding, or covalent attachment. Variation in positions and patterns of combining drugs with NPs results in differential drug delivery capabilities for polymer NPs. The biodegradable poly (lactic-co-glycolic acid) (PLGA) is the commonest among the polymer NPs. It has been reported that using the emulsified solvent diffusion method to package drugs in PLGA NPs can control drug release through mediating inflammatory cell recruitment and inhibiting atherosclerotic plaque instability (Katsuki et al., 2014).

### Dendrimer-based nanoparticles

Dendrimers are highly dendritic polymers with complex three-dimensional structures. The complex structures include host structures and microenvironments, while possessing highly controllable physicochemical parameters. Targeted antibodies, genes, and other bioactive substances are modified on the peripheral groups of the host structures of the dendrimers. Dendrimer-based NPs are regarded as important drug delivery systems relying on several advantages: moderately-sized relative surface areas, high relative molecular masses, low intrinsic viscosities, and good biocompatibilities. Polyamide amine dendrimer is the most frequent selection among the numerous dendrimers used for drug delivery. The slow hydrolysis process of the polyamide amine dendrimer at physiological temperature helps to improve the sustained release of drugs. Dobrovolskaia et al. (2012) showed that platelet aggregation can be inhibited through reducing the positive charge of polyamide amine dendrimer, suggesting that it is very likely to be a potential formulation for anti-thrombosis effects or repairing endothelial injury.

### Metal-based nanoparticles

Metal nanomaterials, including gold, silver, iron, are designed into various sizes, structures, and geometries. Metal NPs are generally smaller in size and exhibit more specific physical, chemical, and biological characteristics. Gold NPs with different shapes like nanospheres, nanorods, and nanoshells are among the most frequently been explored (Yang et al., 2021). Huge possibilities of these metal nanomaterials structures concern their potential use as drug delivery systems, in improving the quality of radiation-based anticancer therapy, in providing photothermal transforming effects for thermal-therapy, and in supporting molecular imaging, as well as being compounds with bactericidal, fungicidal, and anti-viral properties (Sharma et al., 2015; Klębowski et al., 2018).

### Targeting strategies for nanomedicines

**FIGURE 3:**
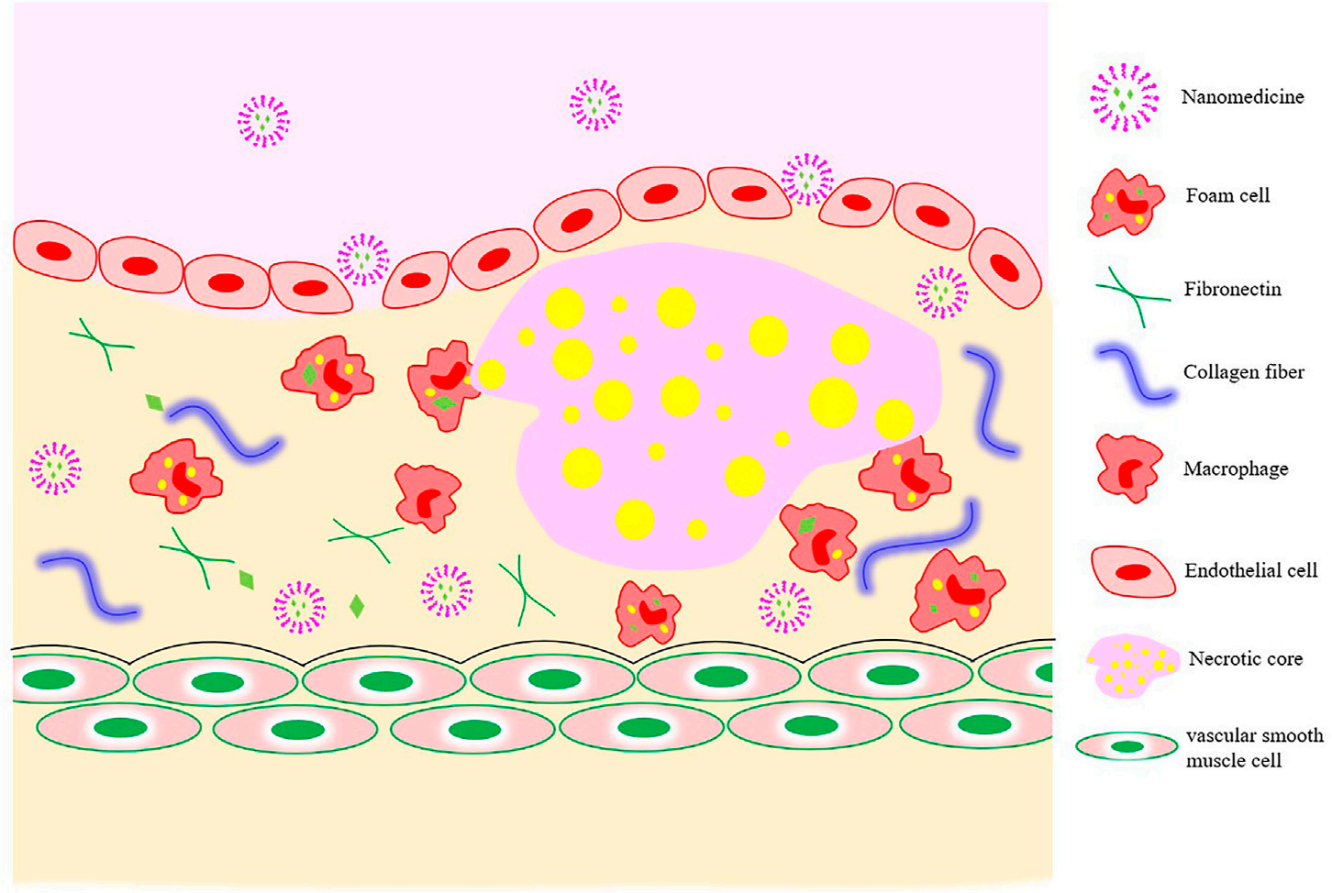
Nanomedicine-based strategies for targeting atherosclerotic plaques.

Because of the tight connection between microvascular endothelial cells in normal tissues and the large relative molecular weights of drug-loaded nanocarriers, their passing through vascular walls is difficult. The increased vascular permeability associated with the development of atherosclerosis provides a pathway for nano-drug delivery from the luminal side to the plaque interior (Figure 3).

The realization of passive targeting mainly depends on the high permeability and high retention of diseased blood vessels. Nanomedicines in the blood circulation are taken up by inflammatory cells (monocytes or macrophages), which then migrate to the inflammatory plaque to exert their functions. When coronary atherosclerosis, thrombosis, or MI occur, the blood vessel lumen is narrowed as the intima of the blood vessels grows laterally and plaque blood flow increases, resulting in an increase in fluid shear force. Nanomaterial-based shear-sensitive drug delivery systems came into existence because of these characteristics. The convex lens-shaped lipid-based NPs prepared by Holme et al. (2012) could not only maintain the structural stability of normal blood vessels, but also release drugs into plaques with structural changes under the action of high blood shear force.

With the in-depth study of specific cells and molecules in the development of atherosclerosis, researchers proposed active targeting strategies based on the pathologic features of atherosclerosis to improve the efficiency of drug targeted delivery to CVDs. Active targeting refers to artificially modifying the function of one or more nanocarrier targets so the drug can precisely reach a specific site to exert its effects. For example, expression of the angiotensin II type 1 receptor (AT1) in peripheral blood is elevated in the early period of myocardial IRI. Dvir et al. (2011) targeted liposomes to infarcted myocardium by modifying ligands that could specifically bind to AT1 on liposomes. With the continuous development of nanomedicines, natural nanomaterials that exhibit excellent inherent targeting properties have aroused strong interest among researchers. One of the most popular endogenous lipid-based NPs is HDL. In addition to removing cholesterol from plaques *via* reverse cholesterol transport, it also works by transporting lipids, proteins, or endogenous miRNA to recipient cells (Kornmueller et al., 2019). In 2019, Jiang et al. (2019) proposed and prepared dual-targeting NPs with recombinant HDL that effectively promoted the rapid regression of plaques. These studies provide new ideas and directions for the prevention and treatment of CVDs.

No matter what targeting strategy is used, including passive targeting, active targeting, intrinsic targeting, or novel strategies for targeting the diseased microenvironment with responsive nanomaterials, the final targeting efficiencies are closely correlated with the biological, physical, and chemical properties of the NPs themselves (Wang et al., 2018). In addition, the targeting efficiencies of nano-formulations are also deeply affected by many different types of objective factors, such as the developmental stage of CVDs, type, location, blood composition, and the wall shear stress.

### Mechanisms of cardiovascular disease

Atherosclerosis is the root cause of most CVDs, and it has three main stages in its evolution. In the first stage, the formation of foam cells is a marker of the onset of atherosclerosis. Under the stimulation of harmful internal and external environmental conditions, the dysregulation of endothelial cells that comprise the capillary walls leads to an increase in permeability to macromolecules. Low-density lipoprotein (LDL) is more likely to cross the vascular wall and accumulate nearby, becoming oxidized LDL (ox-LDL) under the modification of enzymes and reactive oxygen species (Tousoulis et al., 2008; Lobatto et al., 2011). In addition, the expression of chemokines, including monocyte chemotactic protein-1, and inflammatory factors is up-regulated in the damaged vascular endothelial cells. Under the action of the above-mentioned factors, monocytes migrate to the endothelial cells. Monocytes cross the endothelium to differentiate into macrophages driven by adhesion molecules, such as vascular endothelial adhesion molecule (VCAM-1) and intercellular adhesion molecule-1 (ICAM-1). After recognizing and absorbing ox-LDL, macrophages are transformed into foam cells (Bobryshev et al., 2016). In the second stage, vascular smooth muscle cells (VSMCs) under the influence of immune cells and inflammatory factors undergo phenotypic transformation (that is, from contractile phenotype to synthetic phenotype), migrate from the middle membrane to the intima, and begin to proliferate. Some VSMCs absorb ox-LDL and change into VSMC-derived foam cells, and other VSMCs secrete extracellular matrix molecules (such as collagen) to form fibrous caps, inducing neointima formation and vascular remodeling. Eventually, the accumulated foam cells gradually undergo apoptosis or necrosis and turn into the necrotic cores. The final stage is thrombosis or plaque rupture. During this phase, synthetic VSMCs secrete matrix metalloproteinases to degrade the extracellular collagen and thin the fibrous cap. In addition, prolonged oxidative stress promotes further expansion of the necrotic core (Dai et al., 2020; Ge et al., 2020; Park et al., 2020). The enlargement of necrotic core and the formation of new blood vessels may lead to unstable plaque rupture and intravascular thrombosis. Later, persistent arterial spasms ensue and eventually the dreaded lumen occlusions occur (Dai et al., 2016). MI is accompanied by the death of many cardiomyocytes. Even if blood reperfusion is achieved in a very short time, disordered energy metabolism may still occur in the myocardial tissue. In addition, some adverse reactions include the massive accumulation of oxygen free radicals caused by ischemia and hypoxia, Ca^2+^ overload, and inflammatory cascade reactions in myocardial cells, which further aggravate mitochondrial dysfunction, myocardial injury, and even complications from malignant arrhythmia, myocardial fibrosis, or heart failure (Ruytinx et al., 2018).

### Application of nanoparticles in the treatment of atherosclerosis

At present, the non-stimuli-responsive NPs used in the treatment of atherosclerosis are mostly polymeric materials, such as PLGA, cyclodextrin, and chitosan (Psarros et al., 2012). Katsuki et al. reported that pitavastatin delivered by PLGA NPs significantly inhibited plaque rupture when compared with that of pitavastatin alone Kim et al. (2020) invented core-shell NPs based on the self-assembly of 2-hydroxypropyl-β-cyclodextrin and statins. 2-hydroxypropyl-β-cyclodextrin accelerated the removal of cholesterol in the plaque site, and the exchange of the statins in cyclodextrin and cholesterol in the plaque site was driven by host-object affinity. The results indicated that the concentrations of cholesterol and macrophages in plaque were remarkably decreased after the injection of the nanomedicines, which could effectively slow the occurrence of plaques. To further enhance the targeting of NPs, the researchers developed smart, responsive NPs utilizing endogenous stimulation (the specific microenvironment of the plaque site) or exogenous stimulation (e.g., light, ultrasound, and magnetism) (Maruf et al., 2019). In 2017, Dou et al. (2017) chemically modified β-cyclodextrin and encapsulated rapamycin to self-assemble acid-sensitive and reactive oxygen species-sensitive non-pro-inflammatory NPs Compared drug released from non-responsive NPs, drugs released from dual-responsive NPs are more available and achieve better therapeutic effects.

Active targeting NPs are modified with ligand, and the most used ligands are antibodies, peptides, and polymers. Antibodies can be used as target ligands for NPs because of their strong specificity, high affinity, and good stability. Gao et al. (2018) assembled specific antibodies targeting transient receptor potential vanilloid-1 (TRPV1) with CuS NPs to form a photothermal switch (CuS-TRPV1) and activated the TRPV1 channel of VSMCs with the help of a near-infrared laser, resulting in Ca^2+^ influx into VSMCs and the activation of the autophagy pathway. In addition, CuS-TRPV1 can be used in photoacoustic imaging of plaque sites and to accurately control TRPV1 channels, thereby prominently reducing lipid accumulation. Peptide ligands typically consist of 250 amino acids, which are sufficiently small compared with antibodies. They can be loaded into a shallow or hydrophobic binding pocket without compromising specificity or affinity. In addition, they have the advantages of low immunogenicity, simple manufacturing, and easy processing (Kim et al., 2018). Chin et al. (2021) prepared peptide amphiphilic micelles and delivered miR-145 to the plaque site by targeting the chemokine receptor 2 (CCR2) of synthetic VSMCs, thereby modulating the phenotype of VSMCs to slow plaque progression. Li et al. (2020) synthesized cyclodextrin-derived pH-responsive nanoparticles and further modified them with the integrin peptide ligand cRGDfK, which effectively delivered anti-miR33 to macrophages and significantly enhanced the therapeutic effect of the desirable anti-miR33 nanotherapy. Polymers can also serve as ligands, of which hyaluronic acid is the most widely used in atherosclerosis. Hyaluronic acid is an anti-inflammatory and biocompatible polysaccharide that specifically interacts with the CD44 and stabilin-2 receptors expressed by inflammatory and endothelial cells (Liu et al., 2014; Lee et al., 2015; Beldman et al., 2017; Nasr et al., 2020). Ye et al. (2019) successfully fabricated a multimodal and multifunctional NP targeting the class A scavenger receptors, which can be used to arrive at a specific diagnosis and to provide targeted treatment of vulnerable plaques. The designers embedded Fe_3_O_4_ in the shell membranes of NPs and encapsulated perfluorohexane in the core using a double-emulsified solvent evaporation method. Finally, dextran sulfate was adsorbed onto the NPs by electrostatic action. The NPs underwent phase transformation under the influence of low intensity focused ultrasound irradiation, consequently achieving ultrasound imaging, inducing macrophage apoptosis, and relieving plaque burden. Moreover, loaded Fe_3_O_4_ compensated for the deficiency of ultrasound imaging, conducting magnetic resonance imaging of plaques and evaluating vulnerable plaques accurately.

However, traditional NPs still face many challenges in effectively accumulating in atherosclerotic plaques, such as accumulating off-target because of biomarker expression in normal tissues and reduced binding efficiency with limited ligand modification (Xue et al., 2019). At present, researchers have constructed a variety of biomimetic NPs, including recombinant HDL NPs, cell membrane biomimetic NPs, and extracellular vesicle-coated nanoparticles. The interaction between lecithin cholesterol acyltransferase and HDL in the peripheral blood circulation causes drug leakage before reaching the plaque. Furthermore, HDL receptors expressed on the surface of liver cells tend to accelerate HDL accumulation in the liver. Therefore, the most popular biomimetic strategies in recent years are cell membrane and extracellular vesicles. These biomimetic strategies take advantage of the biological function and homing ability of cells or cell components to evade the immune system, extend the circulation time, and implement personalized treatment (Gao et al., 2017).

### Application of nanoparticles in the treatment of hypertension

Most of the main antihypertensive drugs currently used in the clinic have some deficiencies, such as poor water solubility, low bioavailability, and short half-lives. One study showed that compared with conventional dosage forms, the nanoemulsion system had a 2.8-fold increase in the plasma concentration of olmesartan, better antihypertensive efficacy, longer drug maintenance, and a nearly 3-fold reduction in the dose of olmesartan (Alam et al., 2017). Cabrales et al. (2010) used a new platform to prepare a nitric oxide (NO) controlled release systems based on hydrogel/glass hybrid nanoparticles. In addition, NPs served as a system for delivering siRNA and preventing its degradation by endonuclease and exonuclease in blood, serum, and cells. Cationic liposomes made from N-[1-(2,3-dioleoyloxy)]-N-N-N trimethyl ammonium propane (DOTAP) were administered intravenously for 12 days to reduce β1-adrenergic receptor expression and control blood pressure. Moreover, NPs have excellent performance in the early diagnosis of hypertension. An electrochemical immunosensor was sequentially surface-modified using magnetic functionalized diminished graphene oxide conjugated Fe_3_O_4_ NPs; subsequently, the surface of glassy carbon electrode was covered with AuNPs and cortisol antibody. The total amount of cortisol in plasma was detected through competitively combining with antibody sites; the results showed that the amount of cortisol in human plasma samples ranged from 1 to 1,000 ng/ml (Sun et al., 2017). Similarly, other physiological indicators, such as NO, galectin-3, leptin, sodium ions, growth hormone, and inflammatory factors, can be rapidly detected by nanosensors to achieve early diagnosis of hypertension (Madhurantakam et al., 2018).

### Application of nanoparticles in the treatment of pulmonary arterial hypertension

Pulmonary arterial hypertension (PAH) is a highly dangerous and progressive disease characterized by increased pulmonary vascular resistance and elevated pulmonary arterial pressure. The ever-increasing pulmonary vascular resistance leads to pulmonary vasoconstriction and structural remodeling, which, in turn, affects right heart function and ultimately leads to right heart failure or death. The common targeted drugs for the treatment of PAH include prostacyclin (prostaglandin I2), endothelin receptor antagonists, phosphodiesterase type 5 inhibitors, and soluble guanylate cyclase agonists (Nakamura et al., 2017). Bosentan is a selective and competitive endothelin receptor antagonist and the solubility of bosentan NPs is seven times higher than unprocessed bosentan (Ghasemian et al., 2016). Akagi et al. (2016) revealed that PLGA NPs incorporating beraprost observably decreased pulmonary vascular resistance, inhibited pulmonary vascular remodeling, and minimized the occurrence of side effects in a rat model of PAH. Some studies indicated a greater inhibition of pulmonary artery smooth muscle cell proliferation with intratracheal administration of imatinib-incorporated NPs than that with imatinib alone (Akagi et al., 2015). Various nanomedicines including pitavastatin, fasudil, and oligonucleotides have excellent therapeutic effects in inhibiting pulmonary vascular remodeling, reducing pulmonary artery pressure, and improving survival rates (Nakamura et al., 2017).

### Application of nanoparticles in the treatment of myocardial infarction

Because of the low proliferation and limited self-repair ability of cardiomyocytes, cardiac function will decline after acute MI and cannot be restored to the original state. Conventional myocardial reperfusion is not sufficient to repair apoptotic cardiomyocytes and stem cell therapy has become a new treatment method (Madigan and Atoui, 2018). Superparamagnetic iron oxide NPs with unique magnetic properties and good biocompatibility can be used to guide and monitor the therapeutic effect of stem cells on the MI (Zhu et al., 2016). Binsalamah et al. (2011) applied chitosan-alginate NPs to target delivery and sustained release of placental growth factor for the improvement of cardiac function at the site of MI. Leuschner et al. (2011) loaded CCR2-silencing siRNA onto liposomes and injected them intravenously into mice with MI, effectively reducing the aggregation of macrophages and the area of MI. Galagudza et al. (2012) designed silica NPs loaded with adenosine (a prototype cardioprotective agent) to reduce infarct size; in addition, it reduced the hypotension and slow heart rate typically caused by systemic use of adenosine.

It is emphasized that the ischemic myocardium should be reperfused with blood as soon as possible after the occurrence of MI. However, coronary artery reperfusion can sometimes lead to the death of myocardial cells and progressively aggravate tissue damage, which is called myocardial IRI. Ichimura et al. (2016) administered intravenous PLGA NPs loaded with pitavastatin in a porcine myocardial IRI model, and they clearly found that the area of MI was significantly reduced and the left ventricular ejection fraction was distinctly improved 4 weeks later. Furthermore, there was no remarkable impact on blood pressure, heart rate, and biochemical indicators. Yajima et al. (2019) concluded that intravenously injected prostacyclin analog Ono-1301 NPs improved myocardial blood flow in a reperfusion injury model, and the NPs showed selective accumulation and long-term retention in ischemic myocardial tissue. Sayed et al. (2020) reported that a miRNA-loaded dendritic polymeric NPs precisely delivered miRNA into primary rat cardiomyocytes and effectually prevented cardiomyocyte apoptosis resulting from reperfusion. The α-cyclodextrin-based formulations as oxygen nanocarriers can limit IRI by injecting directly into the myocardial wall before starting full blood reperfusion (Femminò et al., 2018). Similarly, Penna et al. (2021) proposed cyclic nigerosyl-nigerose as oxygen nanocarriers, which showed a marked efficacy in controlled oxygenation and effectually protected cellular models from IRI.

Cardiac myocyte death and matrix degradation result in the activation of the innate immune system, and numerous inflammatory factors play a chemotactic role to aggregate neutrophils, monocytes, and macrophages in the damaged myocardium. The continuous stimulation of inflammation further worsens MI and ventricular remodeling. Nakano et al. (2016) have proposed that PLGA NPs doped with irbesartan can antagonize inflammatory monocyte recruitment. They have multiple benefits for alleviating myocardial IRI, diminishing infarct size, and improving left ventricular remodeling. Nicotinamide adenine dinucleotide phosphate oxidase 2 (Nox2), a major source for cardiac reactive oxygen species production, is up-regulated in infarcted myocardium and is closely related to ventricular remodeling. Somasuntharam et al. (2013) demonstrated acid-degradable polyketal particles as delivery vehicles for Nox2-siRNA. After intramyocardial injection into mice with MI, Nox2-siRNA particles not only successfully inhibited the upregulation of Nox2, but also prominently recovered cardiac function. Studies have confirmed that after macrophages phagocytose apoptotic cells, the expression of miRNA-21 is up-regulated and the inflammatory response is alleviated. Bejerano et al. (2018) employed self-assembled NPs *via* Ca^2+^ bridge-mediated hyaluronan-sulfate complexation with nucleic acid to deliver a miRNA-21 mimic to cardiac macrophages after MI. The miRNA-21 NPs induced a phenotype shift from pro-inflammatory to reparative, promoted angiogenesis, and reduced hypertrophy, fibrosis, and cell apoptosis in distal myocardium. Ultimately, left ventricular remodeling was decreased.

Ventricular fibrillation is the leading cause of death in the early stage of MI, especially before admission to the hospital. Amiodarone is currently considered one of the most effective drugs for the treatment of fatal arrhythmia, but there are still limitations in the use of amiodarone in patients with MI. For example, amiodarone may lead to hypotension and non-cardiac death Takahama et al. (2013) utilized liposome-based NPs for targeted delivery of amiodarone. Through *in vivo* intravenous injection in experimental rat models of MI reperfusion, liposome-based NPs not only reduced the mortality associated with malignant arrhythmias, but also attenuated hemodynamic changes induced by amiodarone alone.

### Application of nanoparticles in the treatment of other cardiovascular diseases

As an innovative drug delivery platform, NPs also perform well in the treatment of many other CVDs. NPs targeted delivery of tissue plasminogen activator (t-PA) and other thrombolytic drugs, which played a role in rapid recanalization of occlusive blood vessels, improved the inefficiency of systemic medication, and decreased bleeding and other complications significantly (Torchilin, 2014). Intimal hyperplasia remains a major cause of poor patient outcomes after open vascular reconstructions to treat atherosclerosis. The application of the NPs platform for periadventitial drug delivery may benefit patients undergoing surgical revascularization (Chaudhary et al., 2016). One research team used magnetic NPs to deliver t-PA to the thrombosis area and the drug accumulation at the lesion site was significantly increased through the external magnetic field, requiring less than 1% of the dose of free drug to achieve an effective concentration (Cicha, 2015). Vani et al. (2016) indicated that fullerene NPs considerably decreased the brain IRI; in addition, the enhanced glutathione and superoxide dismutase availably scavenged free radicals and protected brain cells from oxidative damage. Some nano-drug carriers had the ability to cross the blood-brain barrier, which delivered the neuroprotective drug cytidine 5′-diphosphocholine directly into the brain to diminish brain damage caused by ischemia/reperfusion (Panagiotou and Saha, 2015). Evans et al., (2015) developed the polyplex nanocarrier platform to encapsulate vasoactive peptides for alleviation of pathological vasoconstriction. The α-cyclodextrin and α-cyclodextrin nanosponges used as oxygenated nanocarriers release oxygen for a long time and can also be perfused in sufficient solution during organ transportation. The adequate oxygenation may extend the usability time of the explanted organ and promote the postoperative recovery of the transplanted heart (Penna et al., 2022).

### The nanotoxicity on patients with cardiovascular diseases

NPs can be distributed throughout the body by availably translocating in the blood circulation through membrane barriers and affect organs and tissues at both cellular and molecular levels. The interaction between NPs and cells may give rise to nanotoxicity. The narrow size distribution, large surface area to mass ratio, surface properties, charge, dose, and host immunity of NPs were considerably correlated with nanotoxicity. NPs can enter tissues and cells by invading membranes and cause cell damage and toxicity. Contents of oxidative stress (Harrison et al., 2003), inflammation (Libby et al., 2002), mitochondrial DNA damage to the aorta (Ballinger et al., 2000; Ballinger et al., 2002), and damage to vascular endothelial cells (Choksi et al., 2004) have been proposed to cause atherosclerosis. Studies have shown that exposure to NPs exacerbates all these potential triggers (Radomski et al., 2005; Guo et al., 2011; Su et al., 2012), accelerating the progression of atherosclerosis through platelet aggregation and vascular thrombosis (Massberg et al., 2002; Libby et al., 2011).

**FIGURE 4:**
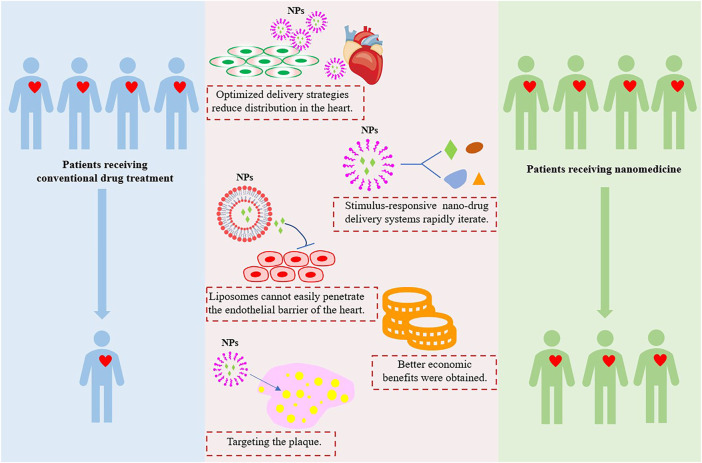
Nanotechnology applications in the advantages of reducing cardiotoxicity. Image adapted from Su et al. (2021).

On the other hand, nanomedicine has attracted great attention, and its applications in CVDs treatment and cardiotoxicity reduction are relatively new and growing. The ideal nanomedicine should be able to target the plaque tissues, penetrate the core of the plaque, and completely remove the plaque without causing systemic damage, especially cardiotoxicity. There are five advantages of nanotechnology in reducing cardiotoxicity (Figure 4). However, the harmful effects of nanocarriers on patients are not fully understood, it is important to further explore the role of NPs in reducing cardiotoxicity in the treatment of CVDs, and long-term detailed toxicity assessments are needed to accurately evaluate the nanotoxicity.

## Conclusion, challenges, and perspectives

**FIGURE 5:**
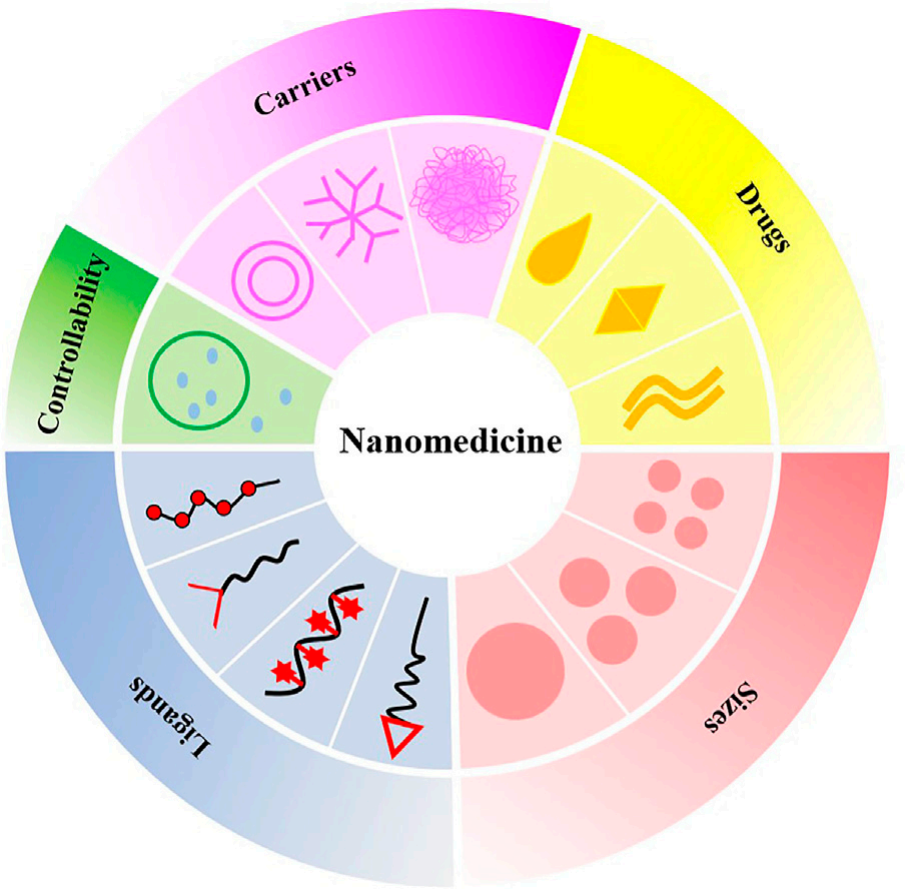
Properties required for ideal nanoparticle.

To summarize, nanomedicine technology in the form of nanocarriers have unique advantages and potential and provide new ideas, approaches, and methods in the diagnosis and treatment of CVDs and a bright prospect for clinicians. Compared with traditional drug delivery methods, nano-drug delivery introduces different ligands into corresponding nanocarriers according to different pathological mechanisms and therapeutic strategies to directly target the lesion site. This strategy more efficaciously targets the atherosclerotic plaque region, increasing drug concentration to improve myocardial blood flow. However, because of the rapid blood flow and the frequent interaction between nanomedicine and numerous blood cells and immune cells and a variety of biomolecules such as chemokines and cytokines, it is difficult to achieve ideal diagnostic and therapeutic expectations. The biomimetic principle is becoming more and more popular in nano-DDSs. Compared with traditional NPs, biomimetic NPs have natural advantages in escaping immune system attack, extending circulation time *in vivo*, and enhancing targeting. Therefore, the combination of traditional NPs and novel biomimetic strategies is an effective treatment. Moreover, cells as nanocarriers are promising by virtue of their strong targeting ability, with some having therapeutic effects of their own. Because of the above NPs design strategy, combination with molecular imaging technology, the construction of integrated diagnosis and treatment NPs will further provide more abundant information for CVDs treatment. Certainly, to maximize the therapeutic efficacy of nanomedicine, more attention should be paid to the structural design, targeting, stability, and safety of NPs in the future (Perioli et al., 2019). Ideal NPs consist of the following parts: ligands that selectively combine with specific molecules, high-capacity drug-loading nanocarriers, the appropriate drug, and controllable drug release (Figure 5). It is also necessary to explore effective drugs for new targets, including how to promote the regeneration of myocardial cells after MI with nano-DDSs.

Nevertheless, the clinical transformation of NPs remains a huge challenge, because the construction of nanotherapeutic systems is complex, the quality control process is cumbersome, and the biosafety needs to be further substantiated. In addition, the interaction between nanoparticles and pathological tissues needs to be fully elucidated and the number of relevant studies in large-scale animal models needs to increase. To accelerate clinical transformation, future research should focus on how to reduce construction complexity and improve therapeutic effectiveness, and efforts should be made to elucidate relevant mechanisms, such as pharmacokinetics, and possible long-term side effects of NPs. Especially in the simplification of NPs, it is necessary to fully exploit the therapeutic effects of nanomaterials themselves, reducing modifications without sacrificing their targeting properties and achieving the integration of diagnosis and treatment as much as possible. It is believed that with the development of different disciplines, the treatment systems based on NPs will bring a new revolution in the treatment of CVDs.

## References

[B1] Akagi S. Nakamura K. Matsubara H. Kondo M. Miura D. Matoba T. (2016). Intratracheal administration of prostacyclin analogue-incorporated nanoparticles ameliorates the development of monocrotaline and sugen-hypoxia-induced pulmonary arterial hypertension. J. Cardiovasc. Pharmacol. 67 (4), 290–298. 10.1097/FJC.0000000000000352 26745002 PMC4827325

[B2] Akagi S. Nakamura K. Miura D. Saito Y. Matsubara H. Ogawa A. (2015). Delivery of imatinib-incorporated nanoparticles into lungs suppresses the development of monocrotaline-induced pulmonary arterial hypertension. Int. Heart. J. 56, 354–359. 10.1536/ihj.14-338 25902888

[B3] Alam T. Khan S. Gaba B. Haider M. F. Baboota S. Ali J. (2017). Nanocarriers as treatment modalities for hypertension. Drug. Deliv. 24 (1), 358–369. 10.1080/10717544.2016.1255999 28165823 PMC8241143

[B4] Baigent C. Baigent L. Blackwell R. Collins J. Emberson J. Godwin R. (2009). Aspirin in the primary and secondary prevention of vascular disease: Collaborative meta-analysis of individual participant data from randomised trials. Lancet 373 (9678), 1849–1860. 10.1016/S0140-6736(09)60503-1 19482214 PMC2715005

[B5] Ballinger S. W. Patterson C. Knight-Lozano C. A. Burow D. L. Conklin C. A. Hu Z. (2002). Mitochondrial integrity and function in atherogenesis. Circulation 106 (5), 544–549. 10.1161/01.cir.0000023921.93743.89 12147534

[B6] Ballinger S. W. Patterson C. Yan C. N. Doan R. Burow D. L. Young C. G. (2000). Hydrogen peroxide- and peroxynitrite-induced mitochondrial DNA damage and dysfunction in vascular endothelial and smooth muscle cells. Circ. Res. 86 (9), 960–966. 10.1161/01.res.86.9.960 10807868

[B7] Bejerano T. Etzion S. Elyagon S. Etzion Y. Cohen S. (2018). Nanoparticle delivery of miRNA-21 mimic to cardiac macrophages improves myocardial remodeling after myocardial infarction. Nano. Lett. 18 (9), 5885–5891. 10.1021/acs.nanolett.8b02578 30141949

[B8] Beldman T. J. Senders M. L. Alaarg A. Pérez-Medina C. Tang J. Zhao Y. (2017). Hyaluronan nanoparticles selectively target plaque-associated macrophages and improve plaque stability in atherosclerosis. Acs. Nano. 11 (6), 5785–5799. 10.1021/acsnano.7b01385 28463501 PMC5492212

[B9] Binsalamah Z. M. Paul A. Khan A. A. Prakash S. Shum-Tim D. (2011). Intramyocardial sustained delivery of placental growth factor using nanoparticles as a vehicle for delivery in the rat infarct model. Int. J. Nanomedicine. 6, 2667–2678. 10.2147/IJN.S25175 22114497 PMC3218580

[B10] Bobryshev Y. V. Ivanova E. A. Chistiakov D. A. Nikiforov N. G. Orekhov A. N. (2016). Macrophages and their role in atherosclerosis: Pathophysiology and transcriptome analysis. Biomed. Res. Int. 2016, 9582430. 10.1155/2016/9582430 27493969 PMC4967433

[B11] Cabrales P. Han G. Roche C. Nacharaju P. Friedman A. J. Friedman J. M. (2010). Sustained release nitric oxide from long-lived circulating nanoparticles. Free. Radic. Biol. Med. 49 (4), 530–538. 10.1016/j.freeradbiomed.2010.04.034 20460149 PMC2903640

[B12] Chaudhary M. A. Guo L. W. Shi X. Chen G. Gong S. Liu B. (2016). Periadventitial drug delivery for the prevention of intimal hyperplasia following open surgery. J. Control. Release. 233, 174–180. 10.1016/j.jconrel.2016.05.002 27179635 PMC4912910

[B13] Chiani M. Shokrgozar M. A. Azadmanesh K. Norouzian D. Mehrabi M. R. Najmafshar A. (2017). Preparation, characterization, and *in vitro* evaluation of bleomycin-containing nanoliposomes. Chem. Biol. Drug. Des. 89 (4), 492–497. 10.1111/cbdd.12869 27637429

[B14] Chin D. D. Poon C. Wang J. Joo J. Ong V. Jiang Z. (2021). miR-145 micelles mitigate atherosclerosis by modulating vascular smooth muscle cell phenotype. Biomaterials 273, 120810. 10.1016/j.biomaterials.2021.120810 33892346 PMC8152375

[B15] Choksi K. B. Boylston W. H. Rabek J. P. Widger W. R. Papaconstantinou J. (2004). Oxidatively damaged proteins of heart mitochondrial electron transport complexes. Biochim. Biophys. Acta. 1688 (2), 95–101. 10.1016/j.bbadis.2003.11.007 14990339

[B16] Cicha I. (2015). Thrombosis: Novel nanomedical concepts of diagnosis and treatment. World. J. Cardiol. 7 (8), 434–441. 10.4330/wjc.v7.i8.434 26322182 PMC4549776

[B17] Cutrone G. Qiu J. Menendez-Miranda M. Casas-Solvas J. M. Aykaç A. Li X. (2019). Comb-like dextran copolymers: A versatile strategy to coat highly porous mof nanoparticles with a peg shell. Carbohydr. Polym. 223, 115085. 10.1016/j.carbpol.2019.115085 31426973

[B18] Dai J. Tian J. Hou J. Xing L. Liu S. Ma L. (2016). Association between cholesterol crystals and culprit lesion vulnerability in patients with acute coronary syndrome: An optical coherence tomography study. Atherosclerosis 247, 111–117. 10.1016/j.atherosclerosis.2016.02.010 26897259

[B19] Dai T. He W. Yao C. Ma X. Ren W. Mai Y. (2020). Applications of inorganic nanoparticles in the diagnosis and therapy of atherosclerosis. Biomater. Sci. 8 (14), 3784–3799. 10.1039/d0bm00196a 32469010

[B20] Dobrovolskaia M. A. Patri A. K. Simak J. Hall J. B. Semberova J. De Paoli Lacerda S. H. (2012). Nanoparticle size and surface charge determine effects of PAMAM dendrimers on human platelets *in vitro* . Mol. Pharm. 9 (3), 382–393. 10.1021/mp200463e 22026635 PMC3624701

[B21] Dou Y. Chen Y. Zhang X. Xu X. Chen Y. Guo J. (2017). Non-proinflammatory and responsive nanoplatforms for targeted treatment of atherosclerosis. Biomaterials 143, 93–108. 10.1016/j.biomaterials.2017.07.035 28778000

[B22] Dvir T. Bauer M. Schroeder A. Tsui J. H. Anderson D. G. Langer R. (2011). Nanoparticles targeting the infarcted heart. Nano. Lett. 11 (10), 4411–4414. 10.1021/nl2025882 21899318 PMC3192253

[B23] Evans B. C. Hocking K. M. Kilchrist K. V. Wise E. S. Brophy C. M. Duvall C. L. (2015). Endosomolytic nano-polyplex platform technology for cytosolic peptide delivery to inhibit pathological vasoconstriction. Acs. Nano. 9 (6), 5893–5907. 10.1021/acsnano.5b00491 26004140 PMC4482421

[B24] Femminò S. Penna C. Bessone F. Caldera F. Dhakar N. Cau D. (2018). α-Cyclodextrin and α-cyclodextrin polymers as oxygen nanocarriers to limit hypoxia/reoxygenation injury: Implications from an *in vitro* model. Polym. (Basel) 10 (2), 211. 10.3390/polym10020211 PMC6414891 30966247

[B25] Fisher M. Moonis M. (2012). Neuroprotective effects of statins: Evidence from preclinical and clinical studies. Curr. Treat. Options. Cardiovasc. Med. 14 (3), 252–259. 10.1007/s11936-012-0174-9 22362392

[B26] Galagudza M. Korolev D. Postnov V. Naumisheva E. Grigorova Y. Uskov I. (2012). Passive targeting of ischemic-reperfused myocardium with adenosine-loaded silica nanoparticles. Int. J. Nanomedicine. 7, 1671–1678. 10.2147/IJN.S29511 22619519 PMC3356166

[B27] Gao J. Wang S. Wang Z. (2017). High yield, scalable and remotely drug-loaded neutrophil-derived extracellular vesicles (EVs) for anti-inflammation therapy. Biomaterials 135, 62–73. 10.1016/j.biomaterials.2017.05.003 28494264 PMC5516786

[B28] Gao W. Sun Y. Cai M. Zhao Y. Cao W. Liu Z. (2018). Copper sulfide nanoparticles as a photothermal switch for TRPV1 signaling to attenuate atherosclerosis. Nat. Commun. 9 (1), 231–310. 10.1038/s41467-017-02657-z 29335450 PMC5768725

[B29] Ge X. Cui H. Kong J. Lu S. Y. Zhan R. Gao J. (2020). A non-invasive nanoprobe for *in vivo* photoacoustic imaging of vulnerable atherosclerotic plaque. Adv. Mater 32 (38), e2000037. 10.1002/adma.202000037 32803803

[B30] Ghasemian E. Motaghian P. Vatanara A. (2016). D-optimal design for preparation and optimization of fast dissolving bosentan nanosuspension. Adv. Pharm. Bull. 6 (2), 211–218. 10.15171/apb.2016.029 27478783 PMC4961979

[B31] Gothwal A. Khan I. Gupta U. (2016). Polymeric micelles: Recent advancements in the delivery of anticancer drugs. Pharm. Res. 33 (1), 18–39. 10.1007/s11095-015-1784-1 26381278

[B32] Guo Y. Y. Zhang J. Zheng Y. F. Yang J. Zhu X. Q. (2011). Cytotoxic and genotoxic effects of multi-wall carbon nanotubes on human umbilical vein endothelial cells *in vitro* . Mutat. Res. 721 (2), 184–191. 10.1016/j.mrgentox.2011.01.014 21296185

[B33] Han H. Bártolo R. Li J. Shahbazi M. A. Santos H. A. (2022). Biomimetic platelet membrane-coated nanoparticles for targeted therapy. Eur. J. Pharm. Biopharm. 172, 1–15. 10.1016/j.ejpb.2022.01.004 35074554

[B34] Harrison D. Griendling K. K. Landmesser U. Hornig B. Drexler H. (2003). Role of oxidative stress in atherosclerosis. Am. J. Cardiol. 91 (3), 7A–11A. 10.1016/s0002-9149(02)03144-2 12645638

[B35] He S. Wu L. Li X. Sun H. Xiong T. Liu J. (2021). Metal-organic frameworks for advanced drug delivery. Acta. Pharm. Sin. B 11 (8), 2362–2395. 10.1016/j.apsb.2021.03.019 34522591 PMC8424373

[B36] Holme M. N. Fedotenko I. A. Abegg D. Althaus J. Babel L. Favarger F. (2012). Shear-stress sensitive lenticular vesicles for targeted drug delivery. Nat. Nanotechnol. 7 (8), 536–543. 10.1038/nnano.2012.84 22683843

[B37] Hossaini Nasr S. H. Rashidijahanabad Z. Ramadan S. Kauffman N. Parameswaran N. Zinn K. R. (2020). Effective atherosclerotic plaque inflammation inhibition with targeted drug delivery by hyaluronan conjugated atorvastatin nanoparticles. Nanoscale 12 (17), 9541–9556. 10.1039/d0nr00308e 32314997 PMC7234819

[B38] Iafisco M. Alogna A. Miragoli M. Catalucci D. (2019). Cardiovascular nanomedicine: The route ahead. Nanomedicine (Lond) 14 (18), 2391–2394. 10.2217/nnm-2019-0228 31456471

[B39] Ichimura K. Matoba T. Nakano K. Tokutome M. Honda K. Koga J. I. (2016). A translational study of a new therapeutic approach for acute myocardial infarction: Nanoparticle-mediated delivery of pitavastatin into reperfused myocardium reduces ischemia-reperfusion injury in a preclinical porcine model. PLoS. One. 11 (9), e0162425. 10.1371/journal.pone.0162425 27603665 PMC5014419

[B40] Jiang C. Qi Z. He W. Li Z. Tang Y. Wang Y. (2019). Dynamically enhancing plaque targeting via a positive feedback loop using multifunctional biomimetic nanoparticles for plaque regression. J. Control. Release. 308, 71–85. 10.1016/j.jconrel.2019.07.007 31295543

[B41] Karunakar G. Patel N. P. Kamal S. S. (2016). Nano structured lipid carrier based drug delivery system. J. Chem. Pharm. Res. 8 (2), 627–643.

[B42] Katsuki S. Matoba T. Nakashiro S. Sato K. Koga J. I. Nakano K. (2014). Nanoparticle-mediated delivery of pitavastatin inhibits atherosclerotic plaque destabilization/rupture in mice by regulating the recruitment of inflammatory monocytes. Circulation 129 (8), 896–906. 10.1161/CIRCULATIONAHA.113.002870 24305567

[B43] Khafaji M. Zamani M. Golizadeh M. Bavi O. (2019). Inorganic nanomaterials for chemo/photothermal therapy: A promising horizon on effective cancer treatment. Biophys. Rev. 11 (3), 335–352. 10.1007/s12551-019-00532-3 31102198 PMC6557961

[B44] Kim H. Kumar S. Kang D. W. Jo H. Park J. H. (2020). Affinity-driven design of cargo-switching nanoparticles to leverage a cholesterol-rich microenvironment for atherosclerosis therapy. Acs. Nano. 14 (6), 6519–6531. 10.1021/acsnano.9b08216 32343121 PMC8543299

[B45] Kim M. Sahu A. Kim G. B. Nam G. H. Um W. Shin S. J. (2018). Comparison of *in vivo* targeting ability between cRGD and collagen-targeting peptide conjugated nano-carriers for atherosclerosis. J. Control. Release. 269, 337–346. 10.1016/j.jconrel.2017.11.033 29175140

[B46] Klębowski B. Depciuch J. Parlińska-Wojtan M. Baran J. (2018). Applications of noble metal-based nanoparticles in medicine. Ijms 19 (12), 4031. 10.3390/ijms19124031 PMC6320918 30551592

[B47] Kornmueller K. Vidakovic I. Prassl R. (2019). Artificial high density lipoprotein nanoparticles in cardiovascular research. Molecules 24 (15), 2829. 10.3390/molecules24152829 PMC6695986 31382521

[B48] Kroll A. V. Fang R. H. Zhang L. (2017). Biointerfacing and applications of cell membrane-coated nanoparticles. Bioconjug. Chem. 28 (1), 23–32. 10.1021/acs.bioconjchem.6b00569 27798829 PMC5471317

[B49] Lee G. Y. Kim J. H. Choi K. Y. Yoon H. Y. Kim K. Kwon I. C. (2015). Hyaluronic acid nanoparticles for active targeting atherosclerosis. Biomaterials 53, 341–348. 10.1016/j.biomaterials.2015.02.089 25890732

[B50] Lee J. J. Saiful Yazan L. S. Che Abdullah C. A. C. (2017). A review on current nanomaterials and their drug conjugate for targeted breast cancer treatment. Int. J. Nanomedicine. 12, 2373–2384. 10.2147/IJN.S127329 28392694 PMC5376210

[B51] Leuschner F. Dutta P. Gorbatov R. Novobrantseva T. I. Donahoe J. S. Courties G. (2011). Therapeutic siRNA silencing in inflammatory monocytes in mice. Nat. Biotechnol. 29 (11), 1005–1010. 10.1038/nbt.1989 21983520 PMC3212614

[B52] Li C. Dou Y. Chen Y. Qi Y. Li L. Han S. (2020). Site‐specific MicroRNA‐33 antagonism by pH‐responsive nanotherapies for treatment of atherosclerosis via regulating cholesterol efflux and adaptive immunity. Adv. Funct. Mat. 30 (42), 2002131. 10.1002/adfm.202002131

[B53] Libby P. Ridker P. M. Hansson G. K. (2011). Progress and challenges in translating the biology of atherosclerosis. Nature 473 (7347), 317–325. 10.1038/nature10146 21593864

[B54] Libby P. Ridker P. M. Maseri A. (2002). Inflammation and atherosclerosis. Circulation 105 (9), 1135–1143. 10.1161/hc0902.104353 11877368

[B55] Liu L. He H. Zhang M. Zhang S. Zhang W. Liu J. (2014). Hyaluronic acid-decorated reconstituted high density lipoprotein targeting atherosclerotic lesions. Biomaterials 35 (27), 8002–8014. 10.1016/j.biomaterials.2014.05.081 24947229

[B56] Lobatto M. E. Fayad Z. A. Silvera S. Vucic E. Calcagno C. Mani V. (2010). Multimodal clinical imaging to longitudinally assess a nanomedical anti-inflammatory treatment in experimental atherosclerosis. Mol. Pharm. 7 (6), 2020–2029. 10.1021/mp100309y 21028895 PMC3345199

[B57] Lobatto M. E. Fuster V. Fayad Z. A. Mulder W. J. (2011). Perspectives and opportunities for nanomedicine in the management of atherosclerosis. Nat. Rev. Drug. Discov. 10 (11), 835–852. 10.1038/nrd3578 22015921 PMC3623275

[B58] Madhurantakam S. Babu K. J. Rayappan J. B. B. Krishnan U. M. (2018). Nanotechnology-based electrochemical detection strategies for hypertension markers. Biosens. Bioelectron. 116, 67–80. 10.1016/j.bios.2018.05.034 29859399

[B59] Madigan M. Atoui R. (2018). Therapeutic use of stem cells for myocardial infarction. Bioeng. (Basel) 5 (2), 28. 10.3390/bioengineering5020028 PMC6027340 29642402

[B60] Martínez-Milla J. Raposeiras-Roubín S. Pascual-Figal D. A. Ibáñez B. (2019). Role of beta-blockers in cardiovascular disease in 2019. Rev. Esp. Cardiol. Engl. Ed. 72 (10), 844–852. 10.1016/j.rec.2019.04.014 31402328

[B61] Maruf A. Wang Y. Yin T. Huang J. Wang N. Durkan C. (2019). Atherosclerosis treatment with stimuli-responsive nanoagents: Recent advances and future perspectives. Adv. Healthc. Mat. 8 (11), e1900036. 10.1002/adhm.201900036 30945462

[B62] Massberg S. Brand K. Grüner S. Page S. Müller E. Müller I. (2002). A critical role of platelet adhesion in the initiation of atherosclerotic lesion formation. J. Exp. Med. 196 (7), 887–896. 10.1084/jem.20012044 12370251 PMC2194025

[B63] McMurray J. J. Packer M. Desai A. S. Gong J. Lefkowitz M. P. Rizkala A. R. (2014). Angiotensin-neprilysin inhibition versus enalapril in heart failure. N. Engl. J. Med. 371, 993–1004. 10.1056/NEJMoa1409077 25176015

[B64] Nakamura K. Matsubara H. Akagi S. Sarashina T. Ejiri K. Kawakita N. (2017). Nanoparticle-mediated drug delivery system for pulmonary arterial hypertension. J. Clin. Med. 6 (5), 48. 10.3390/jcm6050048 PMC5447939 28468233

[B65] Nakamura Y. Mochida A. Choyke P. L. Kobayashi H. (2016). Nanodrug delivery: Is the enhanced permeability and retention effect sufficient for curing cancer? Bioconjug. Chem. 27 (10), 2225–2238. 10.1021/acs.bioconjchem.6b00437 27547843 PMC7397928

[B66] Nakano Y. Matoba T. Tokutome M. Funamoto D. Katsuki S. Ikeda G. (2016). Nanoparticle-mediated delivery of irbesartan induces cardioprotection from myocardial ischemia-reperfusion injury by antagonizing monocyte-mediated inflammation. Sci. Rep. 6 (1), 29601–29614. 10.1038/srep29601 27403534 PMC4939605

[B67] Owens A. T. Brozena S. Jessup M. (2017). Neprilysin inhibitors: Emerging therapy for heart failure. Annu. Rev. Med. 68, 41–49. 10.1146/annurev-med-052915-015509 27686019

[B68] Panagiotou S. Saha S. (2015). Therapeutic benefits of nanoparticles in stroke. Front. Neurosci. 9, 182. 10.3389/fnins.2015.00182 26041986 PMC4436818

[B69] Park J. H. Dehaini D. Zhou J. Holay M. Fang R. H. Zhang L. (2020). Biomimetic nanoparticle technology for cardiovascular disease detection and treatment. Nanoscale. Horiz. 5 (1), 25–42. 10.1039/c9nh00291j 32133150 PMC7055493

[B70] Patra J. K. Das G. Fraceto L. F. Campos E. V. R. Rodriguez-Torres M. D. P. Acosta-Torres L. S. (2018). Nano based drug delivery systems: Recent developments and future prospects. J. Nanobiotechnology. 16 (1), 71–33. 10.1186/s12951-018-0392-8 30231877 PMC6145203

[B71] Penna C. Femminò S. Caldera F. Rubin PedrazzoPedrazzo A. Cecone C. Alfì E. (2021). Cyclic nigerosyl-nigerose as oxygen nanocarrier to protect cellular models from hypoxia/reoxygenation injury: Implications from an *in vitro* model. Int. J. Mol. Sci. 22 (8), 4208. 10.3390/ijms22084208 33921614 PMC8073687

[B72] Penna C. Trotta F. Cavalli R. Pagliaro P. (2022). Nanocarriers loaded with oxygen to improve the protection of the heart to be transplanted. Curr. Pharm. Des. 28 (6), 468–470. 10.2174/1381612827666211109112723 34751111

[B73] Perioli L. Pagano C. Ceccarini M. R. (2019). Current highlights about the safety of inorganic nanomaterials in healthcare. Curr. Med. Chem. 26 (12), 2147–2165. 10.2174/0929867325666180723121804 30033865

[B74] Peters D. Kastantin M. Kotamraju V. R. Karmali P. P. Gujraty K. Tirrell M. (2009). Targeting atherosclerosis by using modular, multifunctional micelles. Proc. Natl. Acad. Sci. U. S. A. 106 (24), 9815–9819. 10.1073/pnas.0903369106 19487682 PMC2689312

[B75] Psarros C. Lee R. Margaritis M. Antoniades C. (2012). Nanomedicine for the prevention, treatment and imaging of atherosclerosis. Nanomedicine 8 (1), S59–S68. 10.1016/j.maturitas.2011.12.01410.1016/j.nano.2012.05.006 22640906

[B76] Radomski A. Jurasz P. Alonso-Escolano D. Drews M. Morandi M. Malinski T. (2005). Nanoparticle-induced platelet aggregation and vascular thrombosis. Br. J. Pharmacol. 146 (6), 882–893. 10.1038/sj.bjp.0706386 16158070 PMC1751219

[B77] Regoli D. Plante G. E. Gobeil F. Jr (2012). Impact of kinins in the treatment of cardiovascular diseases. Pharmacol. Ther. 135 (1), 94–111. 10.1016/j.pharmthera.2012.04.002 22537664

[B78] Ruytinx P. Proost P. Van Damme J. Struyf S. (2018). Chemokine-induced macrophage polarization in inflammatory conditions. Front. Immunol. 9, 1930. 10.3389/fimmu.2018.01930 30245686 PMC6137099

[B79] Sayed N. Tambe P. Kumar P. Jadhav S. Paknikar K. M. Gajbhiye V. (2020). miRNA transfection via poly(amidoamine)-based delivery vector prevents hypoxia/reperfusion-induced cardiomyocyte apoptosis. Nanomedicine (Lond) 15 (2), 163–181. 10.2217/nnm-2019-0363 31799897

[B80] Sharma H. Mishra P. K. Talegaonkar S. Vaidya B. (2015). Metal nanoparticles: A theranostic nanotool against cancer. Drug. Discov. Today. 20 (9), 1143–1151. 10.1016/j.drudis.2015.05.009 26007605

[B81] Smith S. C. Jr Smith A. Collins R. Ferrari D. R. Jr Holmes S. Logstrup D. V. (2012). Our time: A call to save preventable death from cardiovascular disease (heart disease and stroke). Glob. Heart 7 (23), 297–305. 10.1161/CIR.0b013e318267e99f10.1016/j.gheart.2012.08.002 25689941

[B82] Solomon S. D. McMurray J. J. Anand I. S. Ge J. Lam C. S. Maggioni A. P. (2019). Angiotensin-neprilysin inhibition in heart failure with preserved ejection fraction. N. Engl. J. Med. 381 (17), 1609–1620. 10.1056/NEJMoa1908655 31475794

[B83] Somasuntharam I. Boopathy A. V. Khan R. S. Martinez M. D. Brown M. E. Murthy N. (2013). Delivery of Nox2-NADPH oxidase siRNA with polyketal nanoparticles for improving cardiac function following myocardial infarction. Biomaterials 34 (31), 7790–7798. 10.1016/j.biomaterials.2013.06.051 23856052 PMC3766948

[B84] Su L. Han L. Ge F. Zhang S. L. Zhang Y. Zhao B. X. (2012). The effect of novel magnetic nanoparticles on vascular endothelial cell function *in vitro* and *in vivo* . J. Hazard. Mat. 235-236, 316–325. 10.1016/j.jhazmat.2012.08.003 22902133

[B85] Su X. Zhang X. Liu W. Yang X. An N. Yang F. (2021). Advances in the application of nanotechnology in reducing cardiotoxicity induced by cancer chemotherapy. Seminars Cancer Biol. S1044-579X (21), 00215–00217. 10.1016/j.semcancer.2021.08.003 34375726

[B86] Sun B. Gou Y. Ma Y. Zheng X. Bai R. Ahmed Abdelmoaty A. A. A. (2017). Investigate electrochemical immunosensor of cortisol based on gold nanoparticles/magnetic functionalized reduced graphene oxide. Biosens. Bioelectron. 88, 55–62. 10.1016/j.bios.2016.07.047 27499382

[B87] Takahama H. Shigematsu H. Asai T. Matsuzaki T. Sanada S. Fu H. Y. (2013). Liposomal amiodarone augments anti-arrhythmic effects and reduces hemodynamic adverse effects in an ischemia/reperfusion rat model. Cardiovasc. Drugs. Ther. 27 (2), 125–132. 10.1007/s10557-012-6437-6 23344929

[B88] Teymouri M. Mashreghi M. Saburi E. Hejazi A. Nikpoor A. R. (2019). The trip of a drug inside the body: From a lipid-based nanocarrier to a target cell. J. Control. Release. 309, 59–71. 10.1016/j.jconrel.2019.07.027 31340187

[B89] Torchilin V. P. (2014). Multifunctional, stimuli-sensitive nanoparticulate systems for drug delivery. Nat. Rev. Drug. Discov. 13 (11), 813–827. 10.1038/nrd4333 25287120 PMC4489143

[B90] Tousoulis D. Charakida M. Stefanadis C. (2008). Endothelial function and inflammation in coronary artery disease. Postgrad. Med. J. 84 (993), 368–371. 10.1136/hrt.2005.066936 18716016

[B91] Valent P. Groner B. Schumacher U. Superti-Furga G. Busslinger M. Kralovics R. (2016). Paul Ehrlich (1854-1915) and his contributions to the foundation and birth of translational medicine. J. Innate. Immun. 8 (2), 111–120. 10.1159/000443526 26845587 PMC6738855

[B92] Vani J. R. Mohammadi M. T. Foroshani M. S. Jafari M. (2016). Polyhydroxylated fullerene nanoparticles attenuate brain infarction and oxidative stress in rat model of ischemic stroke. EXCLI. J. 15, 378–390. 10.17179/excli2016-309 27540350 PMC4983868

[B93] Wang L. Zhao W. Tan W. (2008). Bioconjugated silica nanoparticles: Development and applications. Nano Res. 1 (2), 99–115. 10.1007/s12274-008-8018-3

[B94] Wang Y. Li L. Zhao W. Dou Y. An H. Tao H. (2018). Targeted therapy of atherosclerosis by a broad-spectrum reactive oxygen species scavenging nanoparticle with intrinsic anti-inflammatory activity. Acs. Nano. 12 (9), 8943–8960. 10.1021/acsnano.8b02037 30114351

[B95] Wang Y. Zhang K. Qin X. Li T. Qiu J. Yin T. (2019). Biomimetic nanotherapies: Red blood cell based core-shell structured nanocomplexes for atherosclerosis management. Adv. Sci. (Weinh) 6 (12), 1900172. 10.1002/advs.201900172 31380165 PMC6662054

[B96] Xue Y. Wu Y. Wang Q. Xue L. Su Z. Zhang C. (2019). Cellular vehicles based on neutrophils enable targeting of atherosclerosis. Mol. Pharm. 16 (7), 3109–3120. 10.1021/acs.molpharmaceut.9b00342 31082253

[B97] Yajima S. Miyagawa S. Fukushima S. Sakai Y. Iseoka H. Harada A. (2019). Prostacyclin analogue-loaded nanoparticles attenuate myocardial ischemia/reperfusion injury in rats. JACC. Basic. Transl. Sci. 4 (3), 318–331. 10.1016/j.jacbts.2018.12.006 31312756 PMC6609885

[B98] Yang J. Jia C. Yang J. (2021). Designing nanoparticle-based drug delivery systems for precision medicine. Int. J. Med. Sci. 18 (13), 2943–2949. 10.7150/ijms.60874 34220321 PMC8241788

[B99] Ye M. Zhou J. Zhong Y. Xu J. Hou J. Wang X. (2019). SR-A-targeted phase-transition nanoparticles for the detection and treatment of atherosclerotic vulnerable plaques. Acs. Appl. Mat. Interfaces. 11 (10), 9702–9715. 10.1021/acsami.8b18190 30785263

[B100] Yusuf S. Joseph P. Rangarajan S. Islam S. Mente A. Hystad P. (2020). Modifiable risk factors, cardiovascular disease, and mortality in 155 722 individuals from 21 high-income, middle-income, and low-income countries (PURE): A prospective cohort study. Lancet 395 (10226), 795–808. 10.1016/S0140-6736(19)32008-2 31492503 PMC8006904

[B101] Zhu K. Li J. Wang Y. Lai H. Wang C. (2016). Nanoparticles-assisted stem cell therapy for ischemic heart disease. Stem Cells Int. 2016, 1–9. 10.1155/2016/1384658 PMC4709699 26839552

